# Intraocular scattering as a predictor of driving performance in older adults with cataracts

**DOI:** 10.1371/journal.pone.0227892

**Published:** 2020-01-14

**Authors:** Sonia Ortiz-Peregrina, Carolina Ortiz, Carlos Salas, Miriam Casares-López, Margarita Soler, Rosario G. Anera

**Affiliations:** Department of Optics, Laboratory of Vision Sciences and Applications, University of Granada, Spain; Tongii University, CHINA

## Abstract

Cataracts can limit a person’s ability to perform vision-dependent tasks safely, affecting the quality of life of older people. This study examines the relationship between visual function and driving, by studying which visual parameters might be important for predicting driving performance in older drivers with and without cataracts, ascertaining whether the objective measurement of intraocular scattering should be considered in assessment procedures for older drivers. This cross-sectional study involved a total of 20 older drivers (10 patients with bilateral cataracts and 10 control subjects). All participants were examined for visual acuity, contrast sensitivity, visual discrimination capacity, and intraocular scattering. Driving performance was also tested using a driving simulator. To study the relationship between visual parameters and driving performance, a correlation analysis and regression model were used. Drivers with cataracts showed a significantly impaired (p<0.05) visual function, with an Objective Scattering Index (OSI) 3.5 times greater than the control group. Driving performance was also significantly worse (p<0.05) in drivers with cataracts, reflected by a notable deterioration in lane keeping. The correlation analysis showed significant associations between driving performance and all the visual parameters studied. Finally, the regression model revealed that the OSI was the best predictor of driving performance, accounting for 51.3% of its variance. Visual function and driving performance are markedly deteriorated when cataracts are present. Our results demonstrate that the objective scattering index (OSI) has a high predictive power when it comes to simulated driving performance in older drivers, both with and without cataracts, suggesting that scatter measurements could be important in helping better understand visual limitations in older drivers.

## Introduction

Elderly people represent the fastest-growing segment of the population in all regions around the world [1] resulting in increased numbers of older drivers on the road. It is therefore expected that, in the next decade, 95% of people aged 65 years or older will be active drivers [2]. Visual impairment becomes significantly more prevalent with increasing age, cataracts being one of the most common eye diseases found in the older population [3]. This condition is typically bilateral, seriously compromises visual acuity and contrast sensitivity, and increases disability glare [4]. Although these symptoms improve after surgery [5], it is common for older drivers to spend long periods of time waiting for cataract surgery [6] for which reason they could drive without having the minimal visual acuity required for a driving licence [7]. This has important implications for road safety, given that it has been proven that drivers with cataracts are approximately 2.5 times more likely to have an at-fault crash, as reported by Owsley et al. [6].

Age-related changes in the eye structures, such as acquired lens opacity, promote a natural increase in intraocular scattering [8, 9], resulting in a veil of straylight on the retina that causes disability glare. Thus, cataract drivers experience an important increase in scattering levels since its onset [10], even when visual acuity remains unaffected, gives rise to vision complaints that compromise driving safety, including night vision disturbances, difficulties in low-sun viewing conditions (i.e., just after dawn or just before sunset), recognition problems, cloudy vision, colour and contrast loss, and difficulties in against-the-light viewing conditions [11, 12].

This vision loss and the consequent functional impairments are evaluated in the traditional clinical vision measurement for driving using the standardised visual acuity test, despite there being evidence that visual acuity is not optimal for predicting driving performance [5, 6, 13] and that contrast sensitivity or glare tests should be included in driver assessment [14, 15] reported that drivers with severe contrast impairment were 8 times more likely to be involved in a crash, demonstrating the importance of contrast sensitivity in traffic safety. On the other hand, although the relationship between disability glare generated by the increase in scattering and driving performance still remains unclear [5, 15], there are studies that highlight the importance of straylight measurements in drivers with cataracts, indicating proposed cut-off values for safe driving [14]. Even so, these cut-off values are mere assumptions made based on prior experience, such as subjects self-reporting dangerous experiences [11, 14] rather than objective evidence of how they impact driving. For this reason, to date, there is still no standardised method or test to measure disability glare in drivers.

The purpose of this study was to examine the relationship between visual function and driving, by studying which visual parameters might be important for predicting driving performance in older drivers, both with and without cataracts, ascertaining whether the objective measurement of intraocular scattering should be considered in assessment procedures for older drivers.

## Materials and methods

### Participants

This cross-sectional study involved a total of 26 participants divided into two groups: cataract and control. The cataract group consisted of 16 bilateral cataract patients, 2 of which withdrew due to scheduling concerns and 4 of which were excluded due to simulator sickness. The final sample consisted of 10 patients with cataracts, ranging in age from 56 to 77 (66.4 ± 6.9 years). The patients with cataracts had no other ocular disease and all had a binocular visual acuity of better than 20/40 or 6/12, the minimal visual acuity required to hold a driving licence in Spain. All diagnosis were made by the same ophthalmologist at the University Hospital San Cecilio of Granada (Spain).Ten controls were also tested, ranging in age from 57 to 71 (63.3 ± 4.1 years); these subjects had normal visual acuity (better than 20/20 or 6/6) and were free of ocular pathologies. All the participants were in good general health, were licenced drivers who drove regularly and complied with legal standards regarding the visual acuity for drivers in Spain.

The study was approved by the Human Research Committee of the University of Granada (180/CEIH/2016). Prior to participating in the study, written informed consent was obtained from each participant in accordance with the Helsinki Declaration. All the participants completed a series of visual tests and an objective driving performance assessment with a simulator. During these visual tests, the participants wore the habitual optical correction they use for driving. In addition to this, we added spherical corrections appropriate to the distance from the participant to the simulator screen.

### Visual function

Visual acuity was measured using the POLA VistaVision® Visual Acuity Chart at 5.5 m (log MAR scale), and contrast sensitivity was measured with the CSV-1000 test (Vector Vision, Ohio, USA) under the recommended viewing conditions (log units) [16]. Visual-discrimination capacity was assessed using the Halo v1.0 software, to quantify any visual disturbances (generated by a central high-luminance stimulus) perceived by the subject under low-illumination conditions. This test provides the visual-disturbance index (VDI), using values from 0 to 1, with higher values indicating a greater contribution of visual disturbances, such as glare or visual halos around luminous stimuli, and therefore a poorer visual discrimination capacity [12]. All measurements were taken binocularly.

In addition, optical quality was measured objectively using the OQAS II (Optical Quality Analysis System II, Visiometrics SL, Tarrasa, Spain) optical device, based on the double-pass technique [17]. This device is useful in older patients for measuring the effect of ocular transparency loss caused by ageing or pathology, thus determining the quality of the retinal image. To do this, we used the OSI (Objective Scatter Index), a parameter that permits intraocular scattering to be objectively quantified. OSI is computed as the ratio of the amount of light within an annular area of 12 and 20 min of arc (inner and outer radii) and that recorded within one minute of arc of the central peak in the acquired double-pass image. For a control subject with no cataracts, normal OSI values are lower than 1.0 (although, in some cases, healthy subjects have results slightly above 1.0); the values are between 1.0 and 2.9 for eyes with early cataracts; and they are greater than 3 for eyes with mature cataracts [18, 19]. However, the mean cut-off value for early cataracts differs between authors [19]. Data was collected from both eyes monocularly, with no pupil dilation, to maintain natural conditions.

### Driving simulator experimental design

Below is a description of the experimental design. The tests were carried out using a driving simulator, following a structure similar to that employed by other authors [20].

#### Driving simulator

The driving simulator consists of a model BC Corona ASI320325R car seat, anchored to the structure of the simulator, and a Logitech G27 Racing Wheel (Logitech International S.A., Lausanne, Switzerland) comprising a steering wheel, six-speed gearbox (plus reverse), and pedals (accelerator, brake, and clutch). SIMAX DRIVING SIMULATOR v4.0.8 BETA software (SimaxVirt, Pamplona, Spain) was used for the driving simulation. The virtual visual reproduction of the road scenario was presented on three 1920x1080 pixel screens (FullHD) with a 180° field of view.

#### Scenario design

Participants drove along a route approximately 10.5 km long. This route, followed in daylight conditions, consisted of two main sections with different degrees of complexity. The first section was a dual carriageway of 4.5 km long. This section involved two lanes of traffic in each direction, a 120 km/h speed limit, moderate traffic, a gentle curve, and no buildings in the surroundings. The second section was a winding mountain road of one-lane single carriageway 6 km long, with variable speed limit of 40 km/h to 90 km/h, no buildings, and moderate traffic. More information on the characteristics of the sections can be found elsewhere [21]. All participants underwent three training sessions with a washout period of 7 days between them. In the training sessions, participants drove the simulator in a similar scenario to that used in the experimental drive (without traffic or pedestrians) in order to avoid the impact of possible learning effects. The participants were instructed to drive as they would normally.

#### Driving measurements

To assess driving performance, we considered the following dependent variables for analyses: mean speed; standard deviation of the lateral position (SDLP); distance travelled invading the opposite lane; total distance travelled outside the lane (i.e., the distance travelled invading the opposite lane plus the distance travelled invading the shoulder); and total time needed to complete the circuit. To obtain an overall measure of driving performance, the Overall Driving Performance Score (ODPS) was calculated. This composite score was obtained as in previous studies [5, 22], by obtaining z-scores for each of the individual driving measurements and calculating a mean z-score for each participant. Z-scores are defined as a measurement of how many standard deviations below or above the group mean, an individual value is. The scores were converted so that positives z-scores represented a better performance than the mean.

The individual variables selected to compute the ODPS were: SDLP, total distance driven outside the lane and total time. For total time, it was established that higher values indicated worse performance, given that these drivers needed more time to detect each stimuli presented on the road, and they had greater difficulty properly completing the route. One previous study included this variable in a similar overall score [23], and other research found that visually impaired drivers tend to need more time to complete the route. Although they drive at slower speeds, this is still not enough to recognise signs, hazards, or pedestrians at the same level as people with no visual impairment, indicating worse driving performance [22, 23].

### Statistical analysis

Statistical analyses were performed using SPSS 24.0 (SPSS Inc., Chicago, IL). Differences between the two groups for all vision and driving performance variables were examined with the independent t-test, and the Mann-Whitney U test when the variables did not present a normal distribution. The main effects of group (cataract vs. control) on driving performance were analysed using generalised linear models with age included as a covariate.

The relationship between visual function and driving performance was first explored with a bivariate correlation analysis, employing Pearson correlations. For variables that did not present a normal distribution, non-parametric Spearman correlations were used. Then, to determine which combination of visual parameters could best predict driving performance, a regression model (with a forward stepwise selection) was employed with the overall driving performance Score (ODPS) as a dependent variable, and visual parameters as independent variables. The level of significance was set at p<0.05.

## Results

### Visual function

For the two groups included in the study, no significant differences were found in age (p = 0.237) or sex distribution (p = 0.280). Table 1 provides the mean data for the visual performance of both groups. As we expected, the t-test revealed significant differences between the groups for all the visual parameters, indicating worse visual performance in the cataract group compared to controls. Moreover, optical quality (i.e., OSI values) also deteriorated significantly when cataracts were present. See Table 1.

**Table 1:** Group mean (±SD) vision performance outcomes for the two groups.

	Control	Cataract	p-value
Binocular VA (log MAR)	-0.06 ± 0.06	0.24 ± 0.06	< 0.001*
Binocular CS (log CS)	1.78 ± 0.17	1.12 ± 0.25	< 0.001*
Binocular VDI	0.23 ± 0.21	0.77 ± 0.25	< 0.001†
OSI	0.97 ± 0.39	3.38 ± 2.26	0.020*

*independent t-test.

† Mann-Whitney U test.

VA, visual acuity; CS, contrast sensitivity; VDI, visual disturbance index; ^|^OSI, objective scatter index.

### Driving performance

Table 2 provides the mean driving performance data for the two groups.

**Table 2:** Group mean (±SD) driving performance outcomes for the two groups.

		Control	Cataract	p-value*
Dual Carriageway	Mean speed (km/h)	113.67 ± 10.49	98.23 ± 14.43	0.014
Mountain Road	SDLP (m)	0.61 ± 0.08	0.74 ± 0.15	0.037
	Distance travelled invading the opposite lane (m)	370.94 ± 185.80	626.84 ± 246.75	0.023
	Total distance travelled outside the lane (m)	436.07 ± 171.38	950.81 ± 494.01	0.006
	Mean speed (km/h)	56.09 ± 2.44	48.98 ± 6.60	0.008
Total Circuit	Total time (s)	694.34 ± 55.60	775.85 ± 93.93	0.030

*independent t-test.

SDLP, standard deviation of the lateral position.

**Fig 1:**
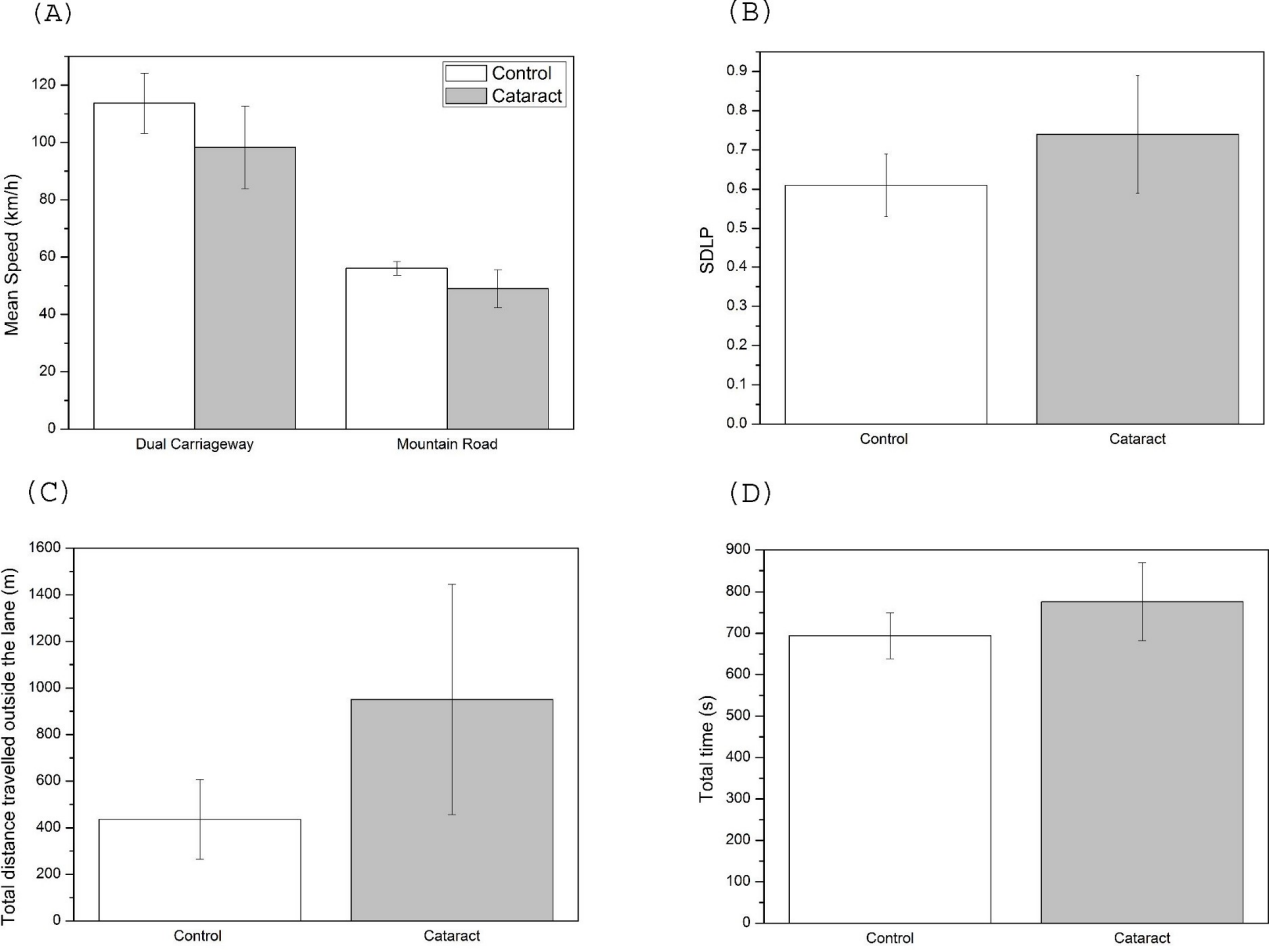
Results of driving performance data for control and cataract groups (mean ± SD): (A) mean speed, (B) SDLP, (C) total distance travelled outside the lane, (D) total time.

For the individual driving parameters, the results revealed a significant main effect of group for the mean speed on both the dual carriageway (χ^2^_1_ = 6.2; p = 0.013) and the mountain road (χ^2^_1_ = 11.2; p = 0.001; Fig 1A). Drivers with cataracts adopted lower speeds than control subjects, with similar reductions seen on both the dual carriageway and mountain road (13.6% and 12.7%, respectively). Cataract drivers showed poorer performance in driving parameters related to lane maintenance, such as a higher SDLP (χ^2^_1_ = 11.2; p = 0.001; Fig 1B), a longer distance driven invading the opposite lane (χ^2^_1_ = 6.4; p = 0.012), and a longer total distance driven outside the lane (χ^2^_1_ = 9.5; p = 0.002; Fig 1C). While the SDLP was 21.3% higher in the cataract group compared to the controls, the total distance travelled outside the lane was more than double for drivers with cataracts (118% higher). The analysis also showed that the cataract group needed 11.7% more time to complete the circuit (χ^2^_1_ = 5.2; p = 0.023; Fig 1D) due to the fact they drove at lower speeds and had more difficulty completing the route. On the other hand, age only presented a significant main effect with regard to mean speed on the dual carriageway (χ^2^_1_ = 5.2; p = 0.022).

Finally, Table 3 shows the z-scores for the individual driving performance parameters, from which the ODPS has been obtained. As can be seen, the cataract group had a significantly worse overall driving score than the control group (χ^2^_1_ = 13.3; p<0.001).

**Table 3:** Z-scores for the individual driving performance parameters employed to calculate the ODPS.

	Control	Cataract
Z score (SDLP)	0.46 ± 0.61	-0.42 ± 1.19
Z score (total distance travelled outside the lane)	0.56 ± 0.39	-0.62 ± 1.12
Z score (total time)	0.47 ± 0.65	-0.65 ± 0.99
ODPS (Mean z-score)*	**0.50 ± 0.42**	**-0.56 ± 0.79**

Notes: Z-scores were converted so that positives values represented a better performance than the mean; ODPS, overall driving performance score.

* p = 0.004 (independent t-test)

### Relationship between vision and driving performance

Table 4 gives the results of the bivariate correlation analysis and shows that all the visual parameters were significantly associated with the individual driving performance parameters. The highest significant correlation was found for the OSI and the total distance driven outside the lane on the mountain road (r = 0.819; p<0.001). Also, the overall driving performance score (ODPS) was significantly predicted by visual acuity, contrast sensitivity, and visual-discrimination capacity (VDI). It can also be observed that there was a significant negative correlation between the ODPS and optical quality (r = -0.716; p = 0.001), since higher OSI values (i.e., greater influence of intraocular scattering) result in worse driving performance (lower ODPS). In fact, OSI was the visual parameter that most strongly correlated with ODPS and the only one that appeared in the final regression model, showing that it alone was the best predictor of driving performance for older drivers.

**Table 4:** Significant correlation coefficients between visual and driving-performance parameters.

		Binocular VA	Binocular CS	Binocular VDI*	OSI
Dual Carriageway	Mean speed (km/h)	-	0.490 (0.028)	-	-0.538 (0.021)
Mountain Road	SDLP (m)	0.484 (0.031)	-0.513 (0.021)	0.467 (0.038)	-
	Total distance driven invading the opposite lane (m)	0.564 (0.015)	-0.519 (0.027)	0.548 (0.019)	0.591 (0.013)
	Total distance driven outside the lane (m)	0.648 (0.003)	-0.652 (0.002)	0.660 (0.002)	0.819 (<0.001)
	Mean speed (km/h)	-0.651 (0.002)	0.711 (<0.001)	-0.551 (0.012)	-0.600 (0.008)
Total circuit	Total time (s)	0.528 (0.017)	-0.624 (0.003)	-	0.550 (0.018)
	ODPS	-0.686 (0.002)	0.686 (0.002)	-0.564 (0.015)	-0.716 (0.001)

Note: p-values are given between parentheses.

* Spearman correlations.

SDLP, standard deviation of the lateral position; ODPS, overall driving performance score; VA, visual acuity; CS, contrast sensitivity; VDI, visual disturbance index; OSI, objective scatter index.

This relationship is expressed by Eq 1, with the standard error indicated in parentheses. The model explains 51.3% of the variation in the overall driving score.

$$\text{ODPS}=0.63-0.27_{\left(0.065\right)}\left(\text{OSI}\right)$$ (1)

## Discussion

In this study, we attempted to investigate the relationship between visual function and driving performance in older drivers with cataracts. First of all, our results from the clinical tests of visual function are in line with previous studies, demonstrating that cataracts seriously diminish visual function [5, 11, 12, 15, 24]. Thus, the cataract group showed a 6-line loss in visual acuity and 0.66 log units in contrast sensitivity. In addition, the OSI was 3.5 times higher in this group, resulting in a greater influence of halos and glare on drivers with cataracts, with a 3-fold increase in VDI.

Additionally, cataracts also negatively impacted driving performance. The cataract group demonstrated worse vehicle control than their non-visually impaired counterparts, as evidenced by the results in the individual measurements of lane keeping. Previous studies have found that older drivers with cataracts fail to adopt an appropriate lane position, having greater difficulties changing lanes or staying in lane when confronted by unexpected events [13, 22]. In fact, lane excursions are the second most common error among older drivers that result in an accident [25]. This lack of ability to properly drive the car within a lane may be due to a progressive deterioration in their visual guidance abilities, affecting the detection of visual cues on the road. However, it may be interesting to study how the visual intervention of road markings affects driving performance behaviour in older drivers. Indeed, some studies state that a comb-shaped marking could solve some traffic problems associated with driving safety [26, 27]. Similarly, general driving performance was also more deteriorated in the cataract group, as shown by the results corresponding to the overall driving performance score. This is in general agreement with other studies of older drivers with real cataracts [5, 22] or simulated cataracts [28]. Crash studies also confirm this finding, as older drivers with cataracts have shown to have 2.5 times more accidents than subjects with no ocular pathologies [6].

The analysis of the relationship between vision and driving showed that all the visual parameters correlated significantly with general driving performance (ODPS), demonstrating the influence of this on driving. Visual acuity and contrast sensitivity show similar associations with ODPS. These parameters have been studied in other works as predictors of driving ability. In fact, visual acuity under optical blur conditions has been shown to have correlation coefficients with driving performance that are comparable to those obtained in our study. Nevertheless, when reduced acuities were due to simulated cataracts, these coefficients decreased by around 0.25 units, showing weaker associations [23]. Apart from this, analyses involving visual acuity for predicting crash data have found only low correlations, typically around 0.1 [29]. This weak correlation could be due to the fact that good visual acuity does not translate into a high level of safety, inasmuch as visual acuity-related driving abilities in tasks such as sign recognition may not be crucial for collision avoidance [30].

On the other hand, our finding of a relatively high correlation between contrast sensitivity and driving performance is in line with other studies, which have shown that the difficulties experienced by older drivers in recognition tasks while driving are predicted by contrast sensitivity [31]. Higgins and Wood [23], studying simulated cataracts, found that the addition of contrast sensitivity to visual acuity significantly increased the percentage of explained variation in several driving performance parameters, including driving time, sign recognition, and hazard avoidance. This is in line with other researchers who have evidenced that drivers suffering cataracts and who had a recent history of crashes are 6 to 8 times more likely to have severe contrast sensitivity impairments [15].

Cataracts contribute to the sensation of glare, diminishing the capacity for discrimination, and revealing a significant negative correlation with driving performance (Table 4). This capacity is key, for example, in night driving, where not detecting peripheral stimuli around the headlights of oncoming traffic could lead to a traffic accident. These results are consistent with previous work where increasing halo size is associated with greater self-reported driving difficulties [32]. Similarly, the study carried out by Kimlin et al. [33], demonstrated a significant correlation between the night-time driving performance of older drivers and halo size.

Although some authors consider that scattering-related measurements (i.e., straylight or disability glare) should be introduced into driver assessment procedures, to date there is still no standardised, uniformly accepted method. The study by van Rijn et al. [10] evaluated the properties of several glare devices, concluding that the measurement of intraocular straylight correlates well with the detrimental effects of glare on perception, being particularly important in subjects with cataracts. Importantly, the work of Ortiz et al. [21] showed a significant correlation between straylight and driving performance, for example in the distance travelled invading the opposite lane, SDLP, and the number of collisions. Moreover, other authors have proposed a cut-off limit for straylight, beyond which driving risk increases. Straylight is a direct consequence of intraocular scattering, but the limit proposed is based on self-reported experiences of danger, and not on objective driving-performance measures [11]. On the other hand, it is necessary to remember that the majority of commercially available glare testers measure the effect of glare on perception (visual acuity or contrast sensitivity). This effect depends a great deal on the specific measurement conditions and it may be that these conditions do not represent the driving conditions when glare is perceived [10]. Therefore, glare measurements are useful in assessing drivers with cataracts, given that they are capable of measuring the component of visual disability introduced by cataract simulators that cannot be detected by acuity alone. This is probably because these measurements may be relatively independent of optical blurring but extremely sensitive to the light-scattering and contrast-reducing properties of the cataract. Indeed, in the study conducted by Higgins and Wood [23] on simulated cataracts, the results indicated that with respect to acuity alone, glare-based tests produced significant increases in the proportion of explained variance for the various driving performance parameters. These studies evaluated the relationship between glare hindrance caused by the optical effect of intraocular scattering and driving performance. However, to the authors’ knowledge, no previous research has objectively investigated intraocular scattering levels and their effect on driving ability. The objective scattering index, which has been studied as a possible predictor of driving performance for the first time in this study, was the visual parameter that most strongly correlated with ODPS, and the only one selected in the regression model, predicting 51.3% of the variance in overall driving scores. This result could be due to the fact that OSI is closely related to the presence of cataracts, and allows a direct and objective quantification of the changes that this generates in the lens. In addition, it is sensitive to the visual changes generated by the cataract, from its onset, while visual acuity or contrast sensitivity may remain unaffected until the disease reaches a more advanced stage [11]. As a result, the level of scattering has been found to change independently of visual acuity in older people, both with and without cataract [34], while contrast sensitivity and visual acuity do correlate [35]. On the other hand, the visual-disturbance index (VDI) makes it possible to evaluate the visual disturbances produced by glare and which are subjectively reported by the observer. It is, therefore, more closely related to the OSI, given that it assesses one of the direct consequences of increased scattered light in the eye media [36].

Thus, the OSI provides a sensitive and specific method for diagnosing cataracts [37, 38], proving to be the most effective optical quality parameter for use in decision-making in cataract surgery [39]. Moreover, it has been proposed as a way of validating a patient’s visual complaints, providing an objective quantification of the visual degradation that actually affects tasks necessary to maintain a good quality of life, such as driving [37].

The relationship between visual and driving performance predicted by our model is similar to that found by Wood [22], who showed that a combination of central motion sensitivity, Useful Field of View (UFOV) scores, contrast sensitivity, and dynamic visual acuity could predict 50% of the variance in overall driving score. However, other studies focusing on individual visual measurements showed that, by themselves, these accounted for less variation in overall driving performance, ranging from 14% to 29% for visual acuity and motion sensitivity, respectively [33]. Furthermore, for crash rates, Hills and Burg [29] found that less than 1% of variance could be accounted for by changes in vision, with visual acuity being the strongest predictor. A better prediction of crash frequency in older drivers was reported by Ball et al. [40], with UFOV accounting for 28% of variance in crash data. This test has proven to be independent of visual acuity, so good visual status does not imply a normal UFOV. The same occurs with intraocular scattering, which increases much faster than visual acuity in ageing and cataractous eyes, both parameters being independent [34]. This shows that the visual acuity measurement does not fully capture the visual capabilities required for driving, and other tests should be included to detect drivers at potential risk. In addition, our results suggest that it is important the test focuses on the potential source of visual reduction, as is the case with the OSI, which directly measures the scattering associated with ageing and/or cataracts.

This study has a number of possible methodological limitations. One is that the driving performance findings are for a driving simulator, making it impossible to capture all the real-world dynamics of driving. However, many studies have demonstrated the validity of driving simulators, indicating that their use provides the most ethical way of conducting studies, ensuring a safe environment for the driver in all situations [41]. Another limitation is the relatively small sample size due to the strict inclusion criteria. Participants for the cataract group had to have bilateral cataracts, but binocular visual acuity of better than 0.5, the minimum required for driving. Furthermore, participants in both groups had to be free of any other ocular disease or surgery, and ocular pathologies become more prevalent with ageing, as does cataract surgery [42]. It is known that older drivers commonly reduce the amount they drive, and even stop driving altogether, this decision often made due to visual impairments like cataracts [43–45]. Our inclusion criteria required regular drivers, limiting the number of eligible cataract patients. In future work it would be interesting to compare groups with different cataract severity, for example, according to the classification proposed by Artal et al. [17] based on OSI values. Another issue resulting in a limited sample size was simulator sickness. These symptoms are common, and more prevalent in older populations while driving a simulator, making it difficult to work with large samples of this population [46]. Moreover, our driving simulator includes a winding mountain road, making the scenario even more challenging with respect to simulator sickness.

In summary, our results show that cataracts can markedly impair some aspects of simulated driving performance, for which reason it could be useful to employ tests that evaluate intraocular scattering levels, something that increases significantly during eye ageing and even more so in the presence of cataracts. In this way, driving performance in older drivers could be largely predicted by the objective scattering index, which accounts for a substantial part of the variance in simulated driving score. It should be borne in mind that this parameter brings together a series of important characteristics and as such is totally objective, avoiding performance errors caused by the diminished cognitive and motor skills of elderly drivers, and being sensitive to the visual disability component (increased scattering), as well as being easy to perform. For this reason, further investigation is necessary to clarify whether including OSI in visual assessments for driving could be a key aspect, helping achieve a better understanding of a driver’s visual status, as well as assist in establishing the moment when visual impairment affects driving ability.

## Supporting information

S1 Table Data underlying the findings described in the manuscript. (PDF)
