# Supplementary material for: Intraocular scattering as a predictor of driving performance in older adults with cataracts
Source: PLoS One. 2020 Jan 14;15(1):e0227892. doi: 10.1371/journal.pone.0227892 (PMC6959599; doi:10.1371/journal.pone.0227892)
Supplement: S1 Table — (PDF) [file pone.0227892.s001.pdf]

S1 Table. Data underlying the findings described in the manuscript.

ID	GROUP*	AGE	VA (Log MAR)	CS (Log CS)	VDI	OSI	MEAN SPEED DUAL CARRIAGEWAY (km/h)	SDLP (m)	DISTANCE TRAVELLED INVADING THE OPPOSITE LANE (m)	TOTAL DISTANCE TRAVELLED OUTSIDE THE LANE (m)	MEAN SPEED MOUNTAIN ROAD (km/h)	TOTAL TIME (s)	ODPS
1	0	65	0.30	0.89	1.00	7.25	102.38	0.70		1988.91	49.57	749.67	-1.11
2	0	72	0.30	0.74	0.83	3.65	78.85	0.84	524.51	841.39	44.14	823.08	-0.87
3	0	56	0.30	1.29	0.80	1.85	95.44	0.56	768.78	784.37	47.99	842.96	-0.22
4	0	59	0.30	0.74	1.00		106.06	0.95	773.25	1032.27	37.62	953.38	-1.79
5	0	61	0.22	1.10	0.31	1.40	109.67	0.72	640.28	793.00	48.56	721.44	-0.15
6	0	73	0.15	1.32	0.53	3.60	79.51	1.00	743.11	1359.82	59.20	758.49	-1.4
7	0	77	0.20	1.22	0.90	6.15	90.77	0.59	941.11	947.81	41.41	858.10	-0.48
8	0	62	0.22	1.17	0.88	1.70	106.84	0.58	489.63	546.52	55.12	679.38	0.54
9	0	72	0.15	1.48	0.43	1.40	88.08	0.64	134.05	263.24	51.47	735.95	0.39
10	0	67	0.30	1.24	1.00		124.65	0.78			54.69	636.05	
11	1	59	0.00	1.78	0.16	0.90	129.31	0.73	431.11	460.82	59.54	618.61	0.48
12	1	65	0.00	1.65	0.21	1.25	110.90	0.55	568.55	581.59	51.23	717.61	0.44
13	1	66	-0.10	2.01	0.20	0.65	116.44	0.54	310.00	317.33	55.97	660.61	0.89
14	1	71	0.00	1.75	0.18	1.15	112.38	0.72	410.00	590.00	56.54	812.00	-0.34
15	1	61	-0.10	1.95	0.81	0.70	120.38	0.64	140.00	290.00	55.89	640.31	0.74
16	1	63	-0.10	1.65	0.24	0.55	127.20	0.67	570.00	640.00	59.65	745.71	0.00
17	1	66	-0.10	1.9	0.16	0.80	103.49	0.65	631.06	659.82	56.65	681.41	0.28
18	1	61	0.00	1.60	0.11	1.25	95.79	0.55	101.47	261.60	56.24	707.60	0.72
19	1	64	-0.15	1.55	0.12	1.80	105.09	0.48	200.82	200.82	55.05	690.61	1.01
20	1	57	-0.08	1.96	0.12	0.65	115.67	0.58	346.40	358.72	54.18	668.91	0.73

*Group: 0 = cataract; 1 = control
